# Sex-specific effects of sympatric mitonuclear variation on fitness in *Drosophila subobscura*

**DOI:** 10.1186/s12862-015-0421-2

**Published:** 2015-07-10

**Authors:** Mihailo Jelić, Göran Arnqvist, Zorana Kurbalija Novičić, Bojan Kenig, Marija Tanasković, Marko Anđelković, Marina Stamenković-Radak

**Affiliations:** Faculty of Biology, University of Belgrade, Studentski trg 16, 11000 Belgrade, Serbia; Animal Ecology, Department of Ecology and Genetics, Uppsala University, Norbyvägen 18D, SE - 752 36 Uppsala, Sweden; Institute for Biological Research “Siniša Stanković”, University of Belgrade, Despot Stefan Blvd. 142, 11000 Belgrade, Serbia; Serbian Academy of Sciences and Arts, Knez Mihailova 35, 11000 Belgrade, Serbia

**Keywords:** Mitochondria, Mitonuclear epistasis, Life history traits, Sexual dimorphism, Selection, Fruit fly

## Abstract

**Background:**

A number of recent studies have shown that the pattern of mitochondrial DNA variation and evolution is at odds with a neutral equilibrium model. Theory has suggested that selection on mitonuclear genotypes can act to maintain stable mitonuclear polymorphism within populations. However, this effect largely relies upon selection being either sex-specific or frequency dependent. Here, we use mitonuclear introgression lines to assess differences in a series of key life-history traits (egg-to-adult developmental time, viability, offspring sex-ratio, adult longevity and resistance to desiccation) in *Drosophila subobscura* fruit flies carrying one of three different sympatric mtDNA haplotypes.

**Results:**

We found functional differences between these sympatric mtDNA haplotypes, but these effects were contingent upon the nuclear genome with which they were co-expressed. Further, we demonstrate a significant mitonuclear genetic effect on adult sex ratio, as well as a sex × mtDNA × nuDNA interaction for adult longevity.

**Conclusions:**

The observed effects suggest that sex specific mitonuclear selection contributes to the maintenance of mtDNA polymorphism and to mitonuclear linkage disequilibrium in this model system.

**Electronic supplementary material:**

The online version of this article (doi:10.1186/s12862-015-0421-2) contains supplementary material, which is available to authorized users.

## Background

Genetic sequence variation within the mitochondrial genome (mtDNA) has by tradition been considered to represent accumulation under the neutral equilibrium model [1, 2]. This view relied largely on the genetics of mtDNA: it is haploid, maternally transmitted and non-recombining. There are relative few mtDNA genes, but these encode fundamental biological processes [3] and any functional variation in such genes should thus swiftly be purged or fixed by natural selection [4]. In recent times, however, a number of studies have shown that the pattern of mitochondrial DNA variation and evolution is often at odds with a neutral model. Evidence of positive selection has been observed in mtDNA sequence data, for example in the form of high *d*_N_/*d*_S_ ratios or correlations between haplotype frequencies and environmental factors [5–9]. Moreover, recent experimental studies that use crossing designs to isolate effects of mtDNA have documented important phenotypic effects of mtDNA variation [10–16]. The maintenance of mtDNA polymorphism, involving functionally non-equivalent haplotypes, may rely upon epistatic interactions between mtDNA and the nuclear genome (nuDNA) (i.e., mitonuclear interactions) [13].

Processes that are governed by mtDNA or hosted by mitochondria (respiration, mitochondrial replication, transcription and even mitochondrial translation) are to a large extent dependent upon products of the nuclear genome and intergenomic mtDNA × nuDNA epistasis should therefore be widespread [17]. Mitonuclear interactions have been observed over several levels of biological organization; from interspecific to intraspecific [18]. Generally, however, mitonuclear incompatibilities tend to increase with genetic distance [19]. Theory predicts that adaptive mtDNA mutations should fix rapidly within populations through positive selection in females [4] and the nuclear genome should co-adapt as a corollary [20], generating population differentiation. It is thus not surprising that many studies have documented mitonuclear interactions between populations while there are much fewer examples of such interactions within populations [18, 21]. Selection on joint mitonuclear genotypes can act to maintain variation in mtDNA [13, 20], but the maintenance of stable mitonuclear polymorphisms within populations seems to require either sex-specific selection or frequency dependent selection [22–25]. Negative frequency dependent selection will occur whenever the relative fitness of a mtDNA haplotype is, for whatever reason, inversely proportional to its frequency [23]. Theory also show that sex specific selection, favoring different mitonuclear types in males and females, can act to maintain mitonuclear polymorphism [13, 25]. Because of the key role that sex-specific mitonuclear fitness interactions may play [13, 24, 25], empirical tests of such sex-specific epistasis are much needed [25].

Experimental studies of mitonuclear fitness interactions within populations have yielded mixed results. Some studies have found no evidence [26, 27] while others have [13, 28]. For example, one study of sympatric cytoplasmic variation on viability in *Drosophila melanogaster* failed to detect any nuclear-cytoplasmic effects, across second chromosome segregation lines [26]. However, another study of the same species [13] revealed sizable nuclear-cytoplasmic effects, across lines carrying different X chromosomes. Yet another study [28] demonstrated sizable sympatric mitonuclear fitness interactions in *D. melanogaster*, but provided limited evidence for sex-specific fitness ranks of mitonuclear variants. Also, studies of *D. subobscura* populations assessing linkage disequilibrium between nuclear markers such as chromosomal inversions or allozymes and mtDNA variability, indicative of selection on mitonuclear genotypes [24, 25], found subtle and seemingly transient disequilibria [29–33]. The possibility that negative frequency dependent selection, presumably generated by habitat heterogeneity, promotes mitonuclear polymorphisms recently received experimental support in a study that monitored changes in haplotype frequencies in 180 laboratory populations of the seed beetle *Callosobruchus maculatus* [34]. Here, the relative fitness of mtDNA haplotypes over 10 generations of experimental evolution was inversely proportional to their starting frequency.

*D. subobscura* is an intriguing model system for the study of genetic variation in mtDNA [29–32, 35–37]. The presence of ubiquitous intra-population variation, with two dominant haplotypes (termed I and II) co-occurring together with typically much rarer endemic haplotypes, in conjunction with weak differentiation between geographically distant populations makes *D. subobscura* an ideal system for understanding the forces that act to maintain within-population variation in mtDNA. Previous studies of natural populations have suggested that both random and adaptive processes are required to account for the observed temporal and geographic distribution of the two dominant haplotypes in this system. A number of studies are indicative of selection on mitonuclear genotypes, and experimental assessments have yielded some evidence for this [16, 30, 31]. Here, we extend this work and ask whether the phenotypic effects of the two dominant haplotypes in *D. subobscura* are sex specific. To achieve this, we create and assay replicated mitonuclear introgression lines.

The construction of mitonuclear introgression lines (MNILs), that harbor different combinations of mitochondrial and nuclear variants, can greatly aid our understanding of the forces that act within populations to maintain genetic variability in mtDNA as it allows the dissection of mitochondrial and nuclear genetic effects on phenotypes [21]. Here, we use MNILs to examine (i) whether segregating genetic variation in mtDNA within populations is functional, (ii) whether phenotypic effects of mtDNA interacts epistatically with segregating nuDNA variation and (iii) whether such effects are sex-specific. We assess a series of key life-history traits independently in males and females. A recent study of the same set of MNILs documented sizeable differences in metabolic rate between flies with different haplotypes, with partly sex-specific effects [27]. However, that study found no significant mitonuclear interactions for adult metabolic rate. In the current study, we provide a detailed dissection of mitochondrial and mitonuclear effects on fitness components such as egg-to-adult developmental time, viability, offspring sex-ratio, adult longevity and resistance to desiccation.

## Results

**Fig. 1:**
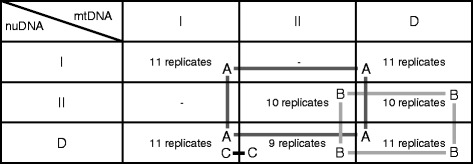
The crossing design used in the mitonuclear introgression experiment. Indicated are also the three crossed modules used for inferential modelling (A, B and C)

**Fig. 2:**
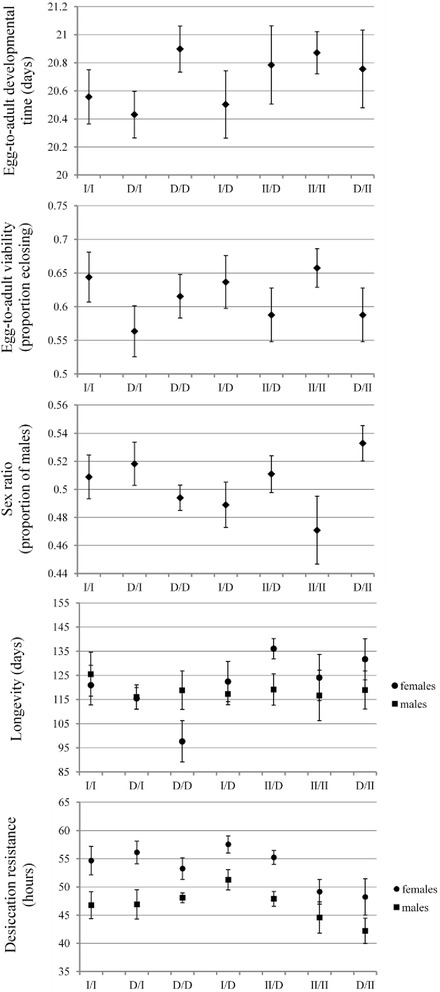
Mean (±S.E.) of various fitness components for MNILs across all mitonuclear cross types. MNILs are denoted as mtDNA / nuDNA

Our crossing design was partially orthogonal, with some combinations of mtDNA × nuDNA missing, and we thus analyzed our data using three different inferential modules (Fig. 1). The first module (A) focused on differences in life history traits between haplotypes I and D, the second (B) on differences between II and D, and the third (C) on differences between I and II. We found no significant effects of mtDNA, nuDNA or their interaction on overall egg-to-adult developmental time or egg-to-adult viability per replicate vial, in any of the three modules (Table 1). There was a tendency for haplotype I to show shorter developmental time and higher viability compared to the other haplotypes (Fig. 2, Additional file 1: Table S1), but this trend was not significant. The addition of vial-specific adult sex-ratio to these models in no case significantly improved model fit to data (*P* > 0.07 in all cases). It is possible that the lack of significant genetic effects on developmental time and viability may, at least in part, be due to laboratory adaptation during introgression.

**Table 1:** The effects of mitochondrial haplotype (mtDNA), nuclear genetic background (nuDNA) and their interaction on egg-to-adult developmental time and overall viability

	Effect	ndf	ddf	F	p
Egg-to-adult developmental time					
Module A	mtDNA	1	40	0.49	0.488
	nuDNA	1	40	1.10	0.301
	mtDNA × nuDNA	1	40	1.87	0.180
Module B	mtDNA	1	36	0.00	0.975
	nuDNA	1	36	0.01	0.929
	mtDNA × nuDNA	1	36	0.10	0.750
Module C	mtDNA	1	18	0.77	0.393
Egg-to-adult viability					
Module A	mtDNA	1	40	1.97	0.169
	nuDNA	1	40	0.29	0.593
	mtDNA × nuDNA	1	40	0.56	0.459
Module B	mtDNA	1	36	0.37	0.549
	nuDNA	1	36	0.39	0.539
	mtDNA × nuDNA	1	36	0.79	0.380
Module C	mtDNA	1	18	0.44	0.514

In terms of the proportion of males produced per vial (Fig. 2, Table 2, Additional file 1: Table S1) haplotype II differed from the others, but mtDNA haplotype effects depended critically upon the nuDNA background. Haplotype II showed a much lower proportion of males compared to the D haplotype in the native II nuDNA background. In the D nuDNA background this effect was reversed, such that haplotype II yielded more males than both haplotypes I and D. Assuming that mtDNA carries no meiotic drive elements [38], such that the primary sex ratio is 1:1 in all lines, this data implies that female juvenile survival was somewhat elevated compared to that in males in flies carrying haplotype II when coexpressed with its native nuDNA. There was also a nuDNA effect, such that flies carrying nuDNA originally associated with mtDNA haplotype I showed a higher proportion of hatching males than those carrying nuDNA associated with the D haplotype. The addition of vial-specific viability to these models in no case significantly improved model fit to data (*P* > 0.24 in all cases, Additional file 2: Table S2). Thus, the observed effects were not due to an overall effect of viability on the proportion of males eclosing.

**Table 2:** The effects of mitochondrial haplotype (mtDNA), nuclear genetic background (nuDNA) and their interaction on the proportion of males hatching

	Fixed term	Wald statistic	ndf	ddf	p
Module A	mtDNA	0.64	1	38.2	0.429
	nuDNA	4.94	1	38.1	***0.032***
	mtDNA × nuDNA	0.03	1	38.2	0.864
Module B	mtDNA	0.16	1	34.4	0.688
	nuDNA	0.66	1	34.8	0.424
	mtDNA × nuDNA	4.45	1	34.7	***0.042***
Module C	mtDNA	4.26	1	256	***0.040***

*P* - <0.05 are bolded and italicized

Our analyses of adult survival (Tables 3 and 4) revealed a series of sex-specific effects. Overall, females lived longer than males did and females were more resistant to desiccation, but the strength of the sex effect on longevity was contingent upon the mitonuclear genotype as evidenced by the sex × mtDNA × nuDNA interactions in modules A and B. Interestingly, the effects of mitonuclear genotype on adult longevity were stronger in females compared to males (Fig. 2, Additional file 1: Table S1). This was corroborated in one-way mixed model ANOVAs of the effect of mitonuclear genotype across all seven genotypes (using MNIL identity as a random effects factor), where effects on longevity were stronger in females (*F*_6,1130_ = 4.22, *P* < 0.001) compared to males (*F*_6,1119_ = 0.21, *P* = 0.974). The same was true, albeit to a lesser extent, for desiccation resistance (*F*_6,1028_ = 2.78, *P* = 0.011 and *F*_6,1085_ = 2.01, *P* = 0.062, respectively). In summary, mtDNA variation had sizeable effects on adult longevity but these effects were contingent upon the nuDNA background and were generally stronger in females compared to males. In contrast, we found no significant mtDNA effects on desiccation resistance in adult flies.

**Table 3:** The effects of mitochondrial haplotype (mtDNA), nuclear genetic background (nuDNA), sex and their interactions on adult longevity

	Source	SS	df	F	p
Module A	Between Subjects				
	mtDNA	2190.95	1	2.76	0.104
	nuDNA	764.90	1	0.96	0.332
	mtDNA × nuDNA	56.53	1	0.07	0.791
	Error	31746.49	40		
	Within Subjects				
	Sex	726.91	1	2.34	0.134
	sex × mtDNA	581.36	1	1.87	0.179
	sex × nuDNA	113.64	1	0.37	0.549
	sex × mtDNA × nuDNA	1410.57	1	4.55	***0.039***
	Error	12414.34	40		
Module B	Between Subjects				
	mtDNA	916.41	1	0.98	0.328
	nuDNA	402.67	1	0.43	0.515
	mtDNA × nuDNA	3268.10	1	3.51	0.069
	Error	34477.14	37		
	Within Subjects				
	Sex	409.00	1	1.07	0.308
	sex × mtDNA	1549.13	1	4.05	0.052
	sex × nuDNA	886.62	1	2.32	0.136
	sex × mtDNA × nuDNA	2195.18	1	5.74	***0.022***
	Error	14149.90	37		
Module C	Between Subjects				
	mtDNA	628.62	1	1.04	0.321
	Error	11512.79	19		
	Within Subjects				
	Sex	1275.99	1	6.72	***0.018***
	sex × mtDNA	365.83	1	1.93	0.181
	Error	3609.85	19		

*P* - <0.05 are bolded and italicized

**Table 4:** The effects of mitochondrial haplotype (mtDNA), nuclear genetic background (nuDNA), sex and their interactions on resistance to desiccation

	Source	SS	df	F	p
Module A	Between Subjects				
	mtDNA	48.14	1	0.65	0.426
	nuDNA	43.90	1	0.59	0.447
	mtDNA × nuDNA	112.46	1	1.51	0.226
	Error	2977.72	40		
	Within Subjects				
	Sex	1124.36	1	70.94	***<0.001***
	sex × mtDNA	0.08	1	0.01	0.943
	sex × nuDNA	44.15	1	2.79	0.103
	sex × mtDNA × nuDNA	8.19	1	0.52	0.477
	Error	634.02	40		
Module B	Between Subjects				
	mtDNA	33.39	1	0.52	0.475
	nuDNA	525.16	1	8.19	***0.007***
	mtDNA × nuDNA	2.93	1	0.05	0.832
	Error	2373.36	37		
	Within Subjects				
	Sex	683.33	1	27.49	***<0.001***
	sex × mtDNA	0.65	1	0.03	0.872
	sex × nuDNA	4.87	1	0.20	0.661
	sex × mtDNA × nuDNA	16.73	1	0.67	0.417
	Error	919.56	37		
Module C	Between Subjects				
	mtDNA	84.59	1	2.07	0.167
	Error	776.88	19		
	Within Subjects				
	Sex	486.55	1	66.43	***<0.001***
	sex × mtDNA	2.99	1	0.41	0.531
	Error	139.17	19		

*P* - <0.05 are bolded and italicized

## Discussion

Our study adds to a handful of studies demonstrating important functional phenotypic differences between sympatric mtDNA haplotypes, *in vivo* [13, 26–28, 39] as well as *in vitro* [40–42]. Collectively, this empirical body of research suggests that the long-standing view of segregating mtDNA variation as being selectively neutral may often be misplaced. In the case of *D. subobscura*, it is highly likely that the phenotypic sex-specific effects on longevity and egg-to-adult survival seen here translates into differences in fitness across mtDNA haplotypes in nature, especially considering the fact that previous studies documented differences in whole-organism metabolic rate [27] and other key life-history traits [36, 43] between flies carrying alternate sympatric mtDNA haplotypes.

Given that selection should act on mtDNA variation within populations of *D. subobscura* [27, 36, 43, 44] one might ask what processes could act to maintain the typical pattern of within-population variation seen in this species; with haplotype I and II co-occurring at fairly similar frequencies. Our results provide three important and relevant insights. First, we found that functional mtDNA effects were contingent upon the nuclear genome with which they were co-expressed. Intergenomic epistatic interactions between the two genomes have previously been documented across populations in many taxa (see [18]) and it has been suggested that such mitonuclear interactions may be important also in *D. subobscura* [16, 27, 30, 31]. Our study confirms that this is indeed the case and it provides a rare example of segregating within-population mitonuclear genetic variation which is demonstrably functional.

Second, we found that mitonuclear genetic effects were sex-specific. This was both strongly suggested in our analysis of adult sex-ratios, presumably reflecting sex-specific egg-adult-survival, and directly demonstrated by the sex × mtDNA × nuDNA interactions seen for adult longevity. Similar sex-specific mitonuclear effects have previously been documented in *D. melanogaster* (e.g., [13, 28]). They are of key importance from a theoretical point of view, as models have shown that sex-specificity of mitonuclear genetic effects can act to maintain stable mtDNA polymorphism [13, 24, 25]. In particular, it has been shown that sex-specific adult viability selection on mitonuclear types promotes the maintenance of mitonuclear polymorphism [25]. Mitonuclear fitness interactions in females are key here; mitonuclear interactions in females are more effective in generating and maintaining mitonuclear polymorphism, simply because females co-transmit mitonuclear combinations while males do not [25]. From this perspective, it is very interesting to note that the mitonuclear genetic effects on adult longevity seen in our study were generally stronger in females than in males. Similarly, a previous study [27] found that mtDNA effects on whole-organism metabolic rate were larger in females than in males in *D. subobscura*. The lack of selection on mtDNA in males can also generate a male-specific genetic load, known as the mother’s curse [45, 46]. This should, however, be particularly evident in male fertility traits [47, 48], which were not assayed here.

Third, the phenotypic effects of nuDNA and mtDNA x nuDNA seen in our study provides indirect evidence for linkage disequilibrium between mtDNA haplotypes and nuclear genes, as the nuclear genomes were classified based on the mtDNA haplotype with which they were co-expressed in the founding population. The founding population should therefore have been characterized by a statistical association between mtDNA and nuDNA. Measurable linkage disequilibrium between mtDNA haplotypes and nuclear genetic markers has previously been demonstrated in *D. subobscura* [31]. Such disequilibria can be generated and maintained by continual mixing of spatially subdivided, and locally adapted populations [49–51] and/or by sex-specific selection on mitonuclear genotypes [24, 25]. We suggest that the sex-specific effects of mitonuclear genotypes seen here, in combination with the fact that the two dominating mtDNA haplotypes (i.e., I and II) occur at roughly equal frequencies in most populations e.g. [30, 32, 52, 53], points to a major role for selection in the maintenance of mitonuclear disequilibria in *D. subobscura*.

Earlier attempts to shed light on the widespread mtDNA polymorphism in natural *D. subobscura* populations assayed similar sets of fitness components as in our study but did not control for nuclear genetic variation [16, 36]. One study [36] found that haplotype II tended to outperform haplotype I in their native nuclear genetic backgrounds, in terms of developmental time, male longevity and resistance to desiccation in both sexes. They were, however, unable to separate mitochondrial and nuclear genetic effects. In another study [16] a single common nuclear genetic background was used and no differences between the two haplotypes were found. Our results (i.e., module C) are consistent with the later study since we generally did not observe significant main differences between haplotypes I and II, although haplotype I tended to outperform haplotype II (Fig. 2, Additional file 1: Table S1). Recently, the authors of a study that assessed metabolic rate suggested that negative frequency dependent selection may contribute to the maintenance of haplotype I and II [27]. This suggestion was based on (i) roughly equal frequencies of haplotypes I and II over the entire species range, (ii) the dynamics of haplotype change in laboratory populations [54] and (iii) recent evidence for negative frequency dependent selection on mtDNA in another insects species [34, 55]. While this may be true, the result of our study suggests an additional mechanism; sex-specific viability selection on mitonuclear genotypes may contribute both to the maintenance of mtDNA polymorphism and to mitonuclear linkage disequilibrium [24, 25].

## Conclusions

We found no significant main differences between sympatric mtDNA haplotypes in *D. subobscura*. Instead, important phenotypic effects of mtDNA variation were contingent upon the nuclear genetic background and were sex-specific. Our findings provide novel support for the possibility that sex-specific mitonuclear selection contributes to the maintenance of mitonuclear genetic variation in this model system.

## Methods

### Construction of mitonuclear introgression lines

The construction of our MNILs is explained in detail elsewhere [27]. Briefly, the experiments reported below were conducted using a set of 73 MNILs of *D. subobscura* representing seven distinct cross types, each with a given combination of either of three specific mtDNA haplotypes (I, II and D) and nuclear genetic backgrounds (Fig. 1). All haplotypes and nuclear genomes originated from a single wild population [32]. Within a particular mtDNA × nuDNA cross type, replicated MNILs were formed from different isofemale strains (IFSs) each of which was founded by a wild-collected female. The exception was the IFS with haplotype D, which was expended in the laboratory to 11 descendant IFSs. Each MNIL derived from two different IFSs and was formed by repeated introgressive backcrossing; 10 virgin females from an IFS with a particular mtDNA haplotype (mitochondrial “Eve’s”) were paired with twice as many males from another IFS with the desired mtDNA haplotype for 12 subsequent generations. Here, then, the paternal contribution provides the nuclear genetic background (classified by the mtDNA haplotype it was originally associated with) during MNIL formation. In theory, more than 99.95 % of the original nuclear genome is thus replaced. To exclude the possibility of mtDNA contamination during introgression, the haplotype integrity of all MNILs were validated at generation 5, 8 and 12 by genotyping a sample of flies from each MNIL. We screened for the presence of *Wolbachia* in all MNILs, by a PCR assay using 16S rDNA *Wolbachia*-specific primers [56] using methods detailed in [52]. We used two different *Drosophila* strains containing *Wolbachia* as positive controls (*D. melanogaster* stock no. 5, Bloomington Stock Centre, *D. simulans*, Riverside strain). These PCR assays were negative for all of our MNILs.

All lines were maintained and all experiments performed under constant laboratory conditions, at 19 °C, approximately 60 % relative humidity, light of 300 lx, and at a photoperiod of 12 h light: 12 h dark. All flies were fed standard *Drosophila* corn-meal medium.

### Fitness assays

#### Egg-to-adult developmental time, viability and sex ratio

2–3 days after eclosion, groups of 15 virgin females and 15 virgin males from the same MNIL were introduced into a 250 ml bottle provided with medium and left to feed, mate and oviposit for 1 week. Females from these bottles were used as focal females, at 9–10 days of adult age. Females were then transferred to oviposition bottles, where watch glasses with live yeast were replaced during three successive days for each oviposition bottle. Eggs were gently removed from the watch glasses with a lancet under a dissection microscope and were transferred to 10 × 3 cm ∅ vials provided with 10 ml of corn medium and live yeast. A total of 13–15 replicates per each MNIL were set up, each consisting of a group of 20 transferred eggs (approximately 21 000 transferred eggs in total). Following the 16th day after egg transfer, all adults that emerged from these vials were counted daily. When all adults had emerged, after day 30, all adults eclosing from each vial were sexed. These assays thus proved data on average egg-to-adult developmental time, egg-to-adult viability (proportion eclosing) and sex-ratio (proportion of males eclosing).

#### Longevity

Longevity assays were performed with virgin flies, collected as they eclosed from their pupa. Here, flies were kept individually in 10 × 3 cm ∅ vials, provided with 5 ml of medium. Each MNIL was represented with 14–16 males and 14–16 females (approximately 2100 individuals in total). Flies were checked daily for survival and medium was replaced once every 2 weeks, as in previous experiments assessing longevity in this species [16, 36]. During medium replacement, we did not observe any cases of dehydration of medium, medium separating from the vial, or mold growing on the food.

#### Resistance to desiccation

Virgin flies at 6–7 days of age were introduced individually into plastic tubes (4 × 0.7 cm ∅). Each tube had 2 small holes (0.5 mm) that allowed air circulation. Flies were checked every hour from the 15th h until they died. As in the longevity experiment, each MNIL was represented with 14–16 individuals of each sex (approximately 2100 individuals in total).

### Statistical analyses

Our experimental design represents a partial orthogonal design with some mtDNA × nuDNA combinations missing and we thus evaluated our results using three different inferential modules (see Fig. 1), each representing a distinct subset of the data that is internally orthogonal and crossed. The first module (A) focussed on differences in life history traits between haplotypes I and D, the second (B) on differences between II and D and the third (C) on differences between I and II. Data on egg-to-adult development time and viability per vial were analysed in general linear mixed models, fitted by REML estimation, using mtDNA and nuDNA as fixed effect factors and MNIL identity as a random effects factor, nested within cross type. Here, data on egg-adult viability was arcsine square root transformed prior to analyses (denominator invariably 20 per observation). We also tested whether the addition of sex ratio per vial improved model fit to data, to account for potential overall sex differences in development time and viability. Data on the sex ratio of eclosing adults (proportion males) was itself analyzed in generalized linear mixed model analogues of these models, using binomial errors and a logit link function with the total number of individuals hatching per vial as the binomial denominator. Here, we additionally tested whether the addition of viability per vial improved model fit to data, to account for overall effects of viability on sex ratio which is shared across genotypes.

For data on longevity and desiccation resistance of individual flies, another classifier (sex) was available. To avoid inflation of the denominator degrees of freedom, each MNIL was here regarded as an experimental unit. Each module was thus analysed by a within-subjects ANOVA (i.e., a partly nested mixed model) of mean values per sex and MNIL, where replicate MNILs were treated as a random effects subject, mtDNA and nuDNA as between subjects factors and sex as a within subjects factor. For all three models, residual distributions were well behaved and in no case did variance ratio tests reveal potential problems with heteroscedasticity, following the removal of a few deviant observations (absolute value of standardized residuals >3.0).

## Availability of supporting data

The data sets supporting the results of this article are included within the article (and its Additional files 3, 4, and 5).

## Additional files

Additional file 1: Table S1. Means (±SE) of fitness components for MNILs that share specific mitochondrial haplotypes (mtDNA) and nuclear genetic backgrounds (nuDNA). Additional file 2: Table S2. The effects of mitochondrial DNA (mtDNA), nuclear genetic background (nuDNA) and their interaction on the proportion of males eclosing after including per-vial viability as a covariate. Additional file 3:
**Row data for egg-to-adult developmental time, viability and sex ratio.**
Additional file 4:
**Row data for longevity.**
Additional file 5
**Row data for resistance to desiccation.**

## Abbreviations

mtDNA: Mitochondrial DNA
nuDNA: Nuclear genetic background
MNIL: Mitonuclear introgression line
