# Supplementary material for: Sex-specific effects of sympatric mitonuclear variation on fitness in Drosophila subobscura
Source: BMC Evol Biol. 2015 Jul 10;15:135. doi: 10.1186/s12862-015-0421-2 (PMC4496845; doi:10.1186/s12862-015-0421-2)
Supplement: Additional file 1: Table S1. — Means (±SE) of fitness components for MNILs that share specific mitochondrial haplotypes (mtDNA) and nuclear genetic backgrounds (nuDNA). [file 12862_2015_421_MOESM1_ESM.doc]

| Table S1 Means (±SE) of fitness components for MNILs that share specific mitochondrial haplotypes (mtDNA) and nuclear genetic backgrounds (nuDNA). | | | | | | | | | | |
| --- | --- | --- | --- | --- | --- | --- | --- | --- | --- | --- |
| FITNESS COMPONENTS | mtDNA | I | | | II | | | D | | |
|  |  |  |  |  |  |  |  |  |  |  |
|  | nuDNA | mean | ± | S.E | mean | ± | S.E | mean | ± | S.E |
| Egg-to-adult developmental time (days) | I | 20.5569 | ± | 0.1940 | - | | | 20.4304 | ± | 0.1657 |
|  | II | - | | | 20.8719 | ± | 0.1508 | 20.7561 | ± | 0.2763 |
|  | D | 20.5033 |  | 0.2403 | 20.7847 | ± | 0.2788 | 20.8983 | ± | 0.1645 |
| Egg-to-adult viability  (proportion eclosing) | I | 0.6439 | ± | 0.0369 | - | | | 0.5635 | ± | 0.0377 |
|  | II | - | | | 0.6575 | ± | 0.0286 | 0.5877 | ± | 0.0398 |
|  | D | 0.6366 | ± | 0.0391 | 0.5877 | ± | 0.0398 | 0.6154 | ± | 0.0324 |
| Sex ratio (proportion of males) | I | 0.5088 | ± | 0.0156 | - | | | 0.5182 | ± | 0.0154 |
|  | II | - | | | 0.4709 | ± | 0.0242 | 0.5329 | ± | 0.0127 |
|  | D | 0.4890 | ± | 0.0162 | 0.5109 | ± | 0.0131 | 0.4940 | ± | 0.0090 |
| Longevity in females (days) | I | 120.9676 | ± | 8.2113 | - | | | 115.4578 | ± | 4.5063 |
|  | II | - | | | 124.0883 | ± | 9.6000 | 131.6900 | ± | 8.5031 |
|  | D | 122.4086 | ± | 8.3147 | 136.0642 | ± | 4.2008 | 97.6784 | ± | 8.5530 |
| Longevity in males (days) | I | 125.4915 | ± | 9.1373 | - | | | 116.0665 | ± | 4.9795 |
|  | II | - | | | 116.6922 | ± | 10.4584 | 118.9809 | ± | 7.8953 |
|  | D | 117.2817 | ± | 4.4117 | 119.1186 | ± | 6.4883 | 118.8472 | ± | 8.0033 |
| Resistance to desiccation in females, (hours) | I | 54.6807 | ± | 2.5151 | - | | | 56.1338 | ± | 1.9997 |
|  | II | - | | | 49.1610 | ± | 2.1874 | 48.2307 | ± | 3.2186 |
|  | D | 57.5476 |  | 1.5374 | 55.2401 | ± | 1.2601 | 53.2587 | ± | 1.9085 |
| Resistance to desiccation in males, (hours) | I | 46.7865 | ± | 2.3890 | - | | | 46.8967 | ± | 2.6057 |
|  | II | - | | | 44.5961 | ± | 2.7757 | 42.2145 | ± | 2.2456 |
|  | D | 51.2667 | ± | 1.8025 | 47.8911 | ± | 1.3157 | 48.0752 | ± | 0.8704 |
