# Supplementary material for: Sex-specific effects of sympatric mitonuclear variation on fitness in Drosophila subobscura
Source: BMC Evol Biol. 2015 Jul 10;15:135. doi: 10.1186/s12862-015-0421-2 (PMC4496845; doi:10.1186/s12862-015-0421-2)
Supplement: Additional file 2: Table S2. — The effects of mitochondrial DNA (mtDNA), nuclear genetic background (nuDNA) and their interaction on the proportion of males eclosing after including per-vial viability as a covariate. [file 12862_2015_421_MOESM2_ESM.doc]

| Table S2 The effects of mitochondrial DNA (mtDNA), nuclear genetic background (nuDNA) and their interaction on the proportion of males eclosing after including per-vial viability as a covariate. | | | | | |
| --- | --- | --- | --- | --- | --- |
|  |  |  |  |  |  |
|  | Fixed term | Wald statistic | ndf | ddf | p |
|  |  |  |  |  |  |
| Module A | Viability | 1.34 | 1 | 325.4 | 0.248 |
|  | mtDNA | 0.86 | 1 | 38.4 | 0.359 |
|  | nuDNA | 4.81 | 1 | 37.4 | **0.035** |
|  | mtDNA × nuDNA | 0.01 | 1 | 37.5 | 0.924 |
|  |  |  |  |  |  |
| Module B | Viability | 0.01 | 1 | 323.7 | 0.906 |
|  | mtDNA | 0.17 | 1 | 34.1 | 0.679 |
|  | nuDNA | 0.63 | 1 | 34.7 | 0.432 |
|  | mtDNA × nuDNA | 4.43 | 1 | 34.2 | **0.043** |
|  |  |  |  |  |  |
| Module C | Viability | 1.37 | 1 | 255 | 0.243 |
|  | mtDNA | 4.03 | 1 | 255 | **0.046** |
|  |  |  |  |  |  |
